# Coupling VIGS with Short- and Long-Term Stress Exposure to Understand the Fiskeby III Iron Deficiency Stress Response

**DOI:** 10.3390/ijms24010647

**Published:** 2022-12-30

**Authors:** Jamie A. O’Rourke, Michelle A. Graham

**Affiliations:** United States Department of Agriculture (USDA), Agricultural Research Service (ARS), Corn Insects and Crop Genetics Research Unit, Ames, IA 50011, USA

**Keywords:** soybean, iron, iron deficiency, tolerance, RNAseq

## Abstract

Yield loss due to abiotic stress is an increasing problem in agriculture. Soybean is a major crop for the upper Midwestern United States and calcareous soils exacerbate iron deficiency for growers, resulting in substantial yield losses. Fiskeby III is a soybean variety uniquely resistant to a variety of abiotic stresses, including iron deficiency. Previous studies identified a MATE transporter (Glyma.05G001700) associated with iron stress tolerance in Fiskeby III. To understand the function of this gene in the Fiskeby III response to iron deficiency, we coupled its silencing using virus-induced gene silencing with RNAseq analyses at two timepoints. Analyses of these data confirm a role for the MATE transporter in Fiskeby III iron stress responses. Further, they reveal that Fiskeby III induces transcriptional reprogramming within 24 h of iron deficiency stress, confirming that like other soybean varieties, Fiskeby III is able to quickly respond to stress. However, Fiskeby III utilizes novel genes and pathways in its iron deficiency response. Identifying and characterizing these genes and pathways in Fiskeby III provides novel targets for improving abiotic stress tolerance in elite soybean lines.

## 1. Introduction

Soybean is the second most valuable crop grown in the US, with 4.44 billion bushels produced in 2021 from over 87 million acres [1]. This makes the United States the world’s leading soybean producer and second only to Brazil in soybean exports [2]. Soybean is a valuable component of human diets, animal feed and biofuel production due to the protein and oil composition of the seed [2]. Much of the soybean production in the United States occurs in the upper Midwestern states, where calcareous soils are prevalent. Plants grown in calcareous soils often suffer from iron deficiency. While iron is plentiful in the soils, various factors including pH, moisture and soil composition can render iron unavailable [3]. Plants have evolved two strategies to obtain iron from the soil. Non-graminaceous plants, such as soybean, are strategy I plants. To increase available Fe^2+^, strategy I plants release protons to acidify the rhizosphere [3]. This reduces Fe^3+^ to Fe^2+^, which can be transported into the plant for use. Strategy I plants also release other compounds, including coumarins, which either chelate Fe^3+^ or reduce Fe^3+^ to Fe^2+^ [4]. If these approaches still do not provide enough iron for the plant, characteristic interveinal yellowing of the leaves (iron deficiency chlorosis (IDC)) and stunted growth phenotypes become evident. The more severe the phenotype, the greater the yield loss at the end of the season. Therefore, mitigating yield loss due to iron deficiency (−Fe) is critically important to farmers, breeders, researchers and private companies. Despite research to alleviate yield loss through seed or foliar treatments, variety selection remains the most important aspect to avoiding −Fe yield loss [5,6].

Fiskeby III is a soybean variety identified from Sweden which exhibits high tolerance to a multitude of abiotic stresses, including −Fe [7,8,9,10,11]. A previous study identified a novel iron deficiency tolerance QTL on chromosome Gm05 in Fiskeby III [12]. Using RNA-seq of Fiskeby III grown in −Fe and +Fe for 14 days, O’Rourke et al. (2021) found that Fiskeby III does not invoke the classic DNA replication/methylation, cell division, and development pathways used by the unrelated iron stress tolerant line Clark under −Fe [13,14]. Instead, Fiskeby III invokes iron stress responses similar to those identified in Arabidopsis including phosphorus homeostasis, transport, and storage. In addition, O’Rourke et al. (2021) used virus-induced gene silencing (VIGS) to screen all the candidate genes located in the Fiskeby III IDC QTL on Gm05. A single gene, *Glyma.05G001700*, conferred an altered phenotype in iron sufficient conditions when silenced. *Glyma.05G001700* encodes a multidrug and toxic compound extrusion (MATE) transporter protein. Glyma.05G001700 is homologous to Arabidopsis DTX14 (AT1G71140), which is known to export the antibiotic norfloxacin [15]. MATE proteins across multiple species are known to regulate biotic stress tolerance [16,17,18]. In Arabidopsis, multiple MATE transporters have been associated with maintaining iron homeostasis. These include *FRD3*, which mediates xylem loading of citrate for transport from roots to shoot, *ELS1* which is iron responsive and regulates leaf senescence, and a golgi-localized MATE, *BCD1*, which reestablishes iron homeostasis in cells when it’s been released by stress or physical damage [19,20,21,22]. We hypothesize the soybean MATE gene, Glyma.05G001700, plays an important role in the soybean iron deficiency stress tolerance response.

**Figure 1:**
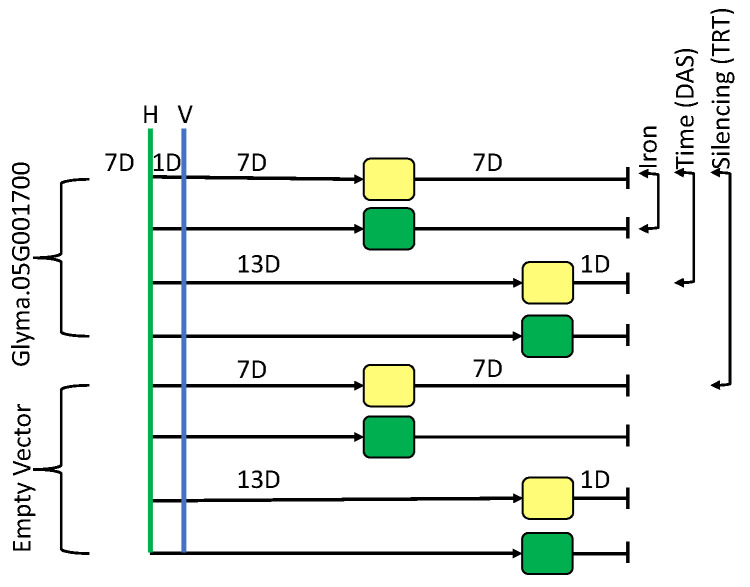
Experimental Design. Soybean plants were germinated in vermiculite. At 7 days (7D) after planting, plants were transferred to a hydroponic system, denoted by H and green vertical line. After 24 h in hydroponics, plants were rub-inoculated with VIGS virus containing either empty vector or vector to silence Glyma.05G001700, denoted by V and vertical blue line. Plants were grown in optimal hydroponic conditions for either 7D or 13D. Half of plants moved at each timepoint were transferred to new sufficient (green) or iron deficient (yellow) conditions. Examples of the three types of comparisons made are denoted on the right.

Previous work by our group has shown soybean gene expression can change within 30 min of stress exposure [23]. Additional studies have found DEGs are often unique to specific timepoints and genotypes, establishing a need to conduct gene expression analyses in multiple genotypes and timepoints [23,24,25,26]. These responses are in stark contrast to Arabidopsis which does not begin to respond to iron stress until sometime between 1 and 6 h after stress exposure, with canonical iron genes changing between 4 and 8 h after stress exposure [27,28]. Findings from our earlier publication indicate that Fiskeby III might function more like Arabidopsis than the soybean line Clark. We examined gene expression in Glyma.05G001700-silenced and control plants after one and seven days of −Fe stress (Figure 1) to better understand the initial stages of the Fiskeby III iron stress response. We expect by comparing Glyma.05G001700-silenced and control plants we will identify novel genes and networks involved in the Fiskeby III iron stress response. These findings will provide novel targets for plant breeders developing IDC-tolerant lines.

## 2. Results

The experiment was designed to measure iron stress responses in either VIGS_Glyma.05G1700 or VIGS_EV (empty vector control) infected Fiskeby III leaves and roots exposed to either one day or seven days of −Fe stress (Figure 1). Iron stress treatments were timed to ensure all plants were the same age at the time of harvest, but after experiencing different durations of −Fe stress. The design of this experiment allowed us to investigate gene expression changes while considering three different factors. The most obvious factor is the effect of silencing *Glyma.05G001700*. In analyses examining the silencing effect, all comparisons are VIGS_Glyma.05G001700 vs. VIGS_EV grown under the same conditions. Second, the iron treatment effect; the same VIGS treatment (VIGS_Glyma.05G001700 or VIGS_EV at 1D or 7D) in +Fe vs. −Fe. Third, the effect of time; the same VIGS treatment (VIGS_Glyma.05G001700 or VIGS_EV) under either +Fe or −Fe conditions, but identifying differences in expression between 1D vs. 7D of −Fe stress. Combining the findings from these three factors provides more detailed insights into the Fiskeby III iron stress response than each factor alone.

### 2.1. Effect of Silencing (VIGS_Glyma.05G001700 vs. VIGS_EV)

**Figure 2:**
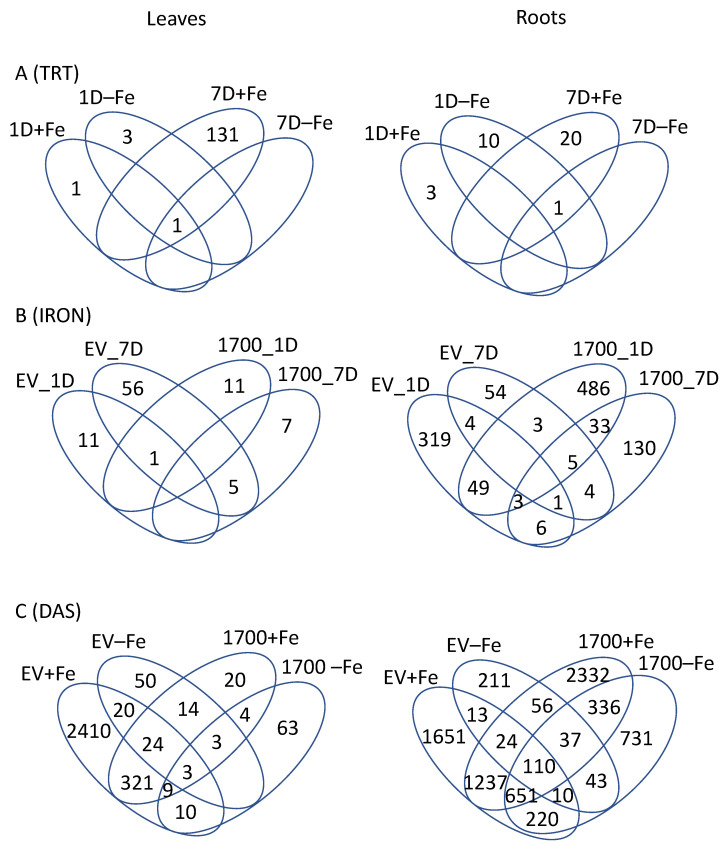
Distribution of differentially expressed genes. Venn diagrams detailing the distribution of statistically significant (FDR < 0.05) differentially expressed genes (DEGs) in each of the three analyses; treatment (**A**, VIGS_Glyma.05G001700 vs. VIGS_EV), iron status (**B**, Fe+ vs. Fe−), and days after stress (**C**, 7D vs. 1D) in leaves (left column) and roots (right column).

**Figure 3:**
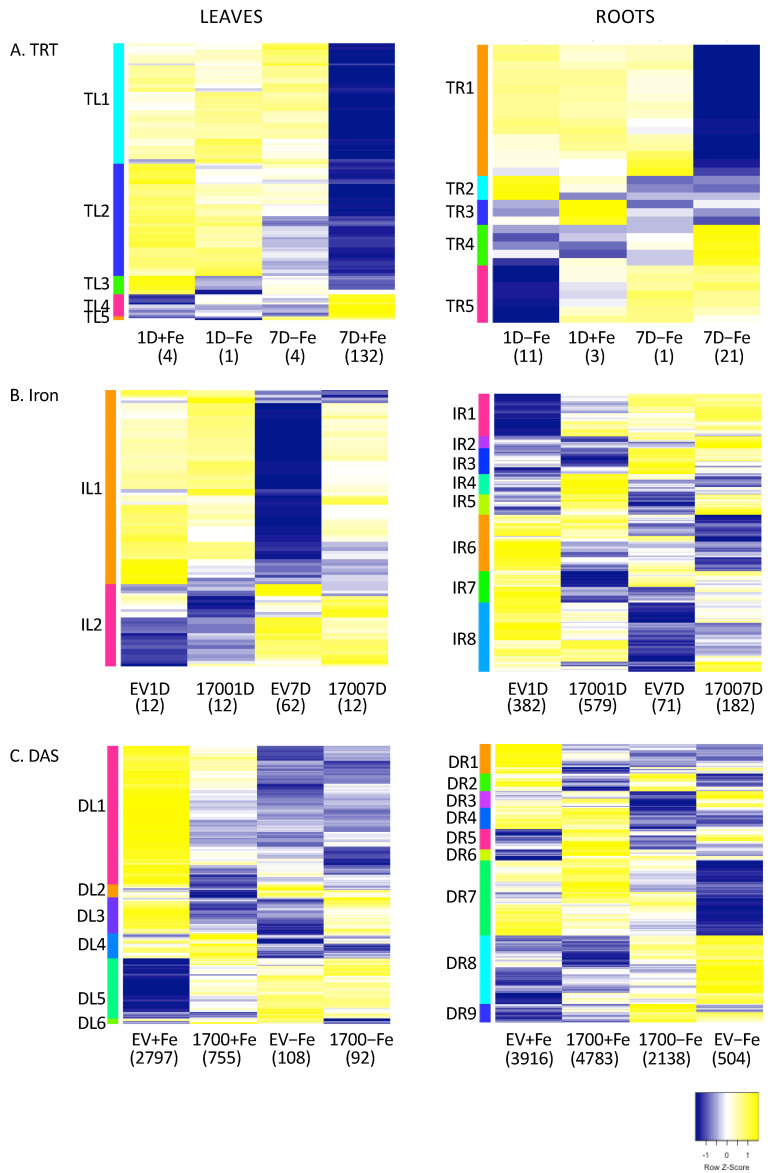
Heatmaps of differentially expressed genes. Z-scores of differentially expressed genes identified from the three analyses (**A**) treatment (VIGS_Glyma.05G001700 vs. VIGS_EV), (**B**) iron status, and (**C**) days of stress (1D vs. 7D). Heatmaps for DEGs identified in leaves are on the left and roots are on the right. Hierarchical clustering was performed in R to identify unique expression clusters, these are denoted to the left of each heatmap by both colored bars and a three-letter code corresponding to either treatment (T), iron status (I) or days of stress (D), tissue (Leaf (L) or Root (R)) and the cluster number. Number of DEGs identified in each comparison is provided in parentheses below the comparison ID. Expression of all DEGs identified in each analysis is represented even if a gene is not statistically differentially expressed in a specific comparison. Negative Z-scores are blue, positive Z-scores are yellow.

Comparing leaves and roots of VIGS_Glyma.05G001700 to VIGS_EV at each treatment and timepoint revealed very few differentially expressed genes (DEGs) (Figure 2A and Tables S1 and S2). In leaves, the majority of the DEGs (132) were identified at 7D in +Fe-grown plants with 120 DEGs up-regulated and 12 DEGs down-regulated. It is notable that there are 10 times as many genes up-regulated as down-regulated. A single gene, *Glyma.19G001500*, was differentially expressed in all four VIGS_Glyma.05G001700 vs. VIGS_EV comparisons in leaves and three of the four comparisons in roots. In all comparisons, silencing *Glyma.05G001700* causes significant up-regulation of *Glyma.19G001500*. *Glyma.19G001500* is homologous to Arabidopsis gene AT3G23590 (*REF4/Medd33a/Med5a/b*), a member of the mediator transcriptional coactivator complex [29,30,31,32,33]. In addition, in leaves, a rubisco gene was up-regulated at 1D+Fe, while a Bax inhibitor protein known to be involved in cell death suppression in response to biotic and abiotic stresses was down-regulated at 1D−Fe [34]. The remaining 131 DEGS from 7D+Fe leaves included seven MYB Transcription Factors (TFs) and genes involved in fatty acid biosynthesis. The 136 DEGs organized into five expression clusters (Figure 3A), though the majority of the genes were in clusters TL1 and TL2, which have very similar expression patterns. To determine if expression clusters could be linked with specific biological functions, we conducted GO term enrichment (Corrected *p* < 0.05) of the genes associated with specific expression clusters. Cluster TL1 corresponded to a single significantly overrepresented GO term (Table S3), alternative respiration (GO:0010230), while TL2 was significantly (Corrected *p* < 0.05) overrepresented with the GO terms very-long chain fatty acid metabolism (GO:0000038) and wax biosynthesis (GO:0010025) (Table S3). No significant GO terms were identified for clusters TL3-TL5.

In roots, the pattern of DEGs in response to VIGS treatment was the same as in leaves, three genes were down-regulated at 1D+Fe, 11 genes were differentially expressed (DE) at 1D−Fe, 21 genes were DE at 7D+Fe and a single gene was DE at 7D−Fe (Figure 2A, Table S2). As in leaves, more genes were up-regulated (17) than down-regulated (4) in the root 7D+Fe comparison. Aside from *Glyma.19G001500*, which was DE in all but the 1D+Fe comparison, no other DEGs were shared by multiple comparisons (Figure 2A). Like leaves, the 34 DEGs are organized into five expression clusters (Figure 3A), with TR1 and TR4 associated with 7D+Fe expression and TR5 associated with 1D−Fe expression. No significant GO terms were associated with TR1-TR5.

### 2.2. Effect of Iron Treatment (+Fe vs. −Fe)

Comparing plants treated with the same VIGS vector (either VIGS_EV or VIGS_Glyma.05G001700) but grown in either +Fe vs. −Fe (Figure 2B, Tables S4 and S5) revealed more DEGs than the silencing effect analyses (Figure 2A). While all plants in this study were infected with the bean pod mottle virus (BPMV), plants infected with the VIGS_EV vector should respond similarly to uninfected plants. In leaves, most DEGs were identified in VIGS_EV after 7D of −Fe stress (62 DEGs) with the remaining comparisons all identifying 12 DEGs with little overlap (Figure 2B and Table S3). This is evidenced by the heatmap generated from expression profiles (Figure 3B). Cluster IL1 is almost exclusively DEGs from EV7D while cluster IL2 is comprised mainly of DEGs from the other comparisons. GO analysis of cluster IL2 identified six significantly overrepresented GO terms associated with phosphate homeostasis mechanisms (Table S3). One notable gene in leaves is *Glyma.17g140400*, which was differentially expressed in both VIGS_EV_1D, VIGS_EV_7D, and in VIGS_ Glyma.05G001700_1D. *Glyma.17G140400* encodes a lipid transfer protein, the Arabidopsis homolog (At2G45180, DRN1) is involved in basal defense responses, responding to changing reactive oxygen species levels [35]. Multiple proteins in this family are involved in salt stress tolerance, with DRN1 suppressed by salt stress and overexpression enhancing salt tolerance [35]. In our experiment, *Glyma.17G140400* expression was suppressed after 1D−Fe stress but increased after 7D−Fe stress. The increased expression after 7D−Fe stress in Fiskeby III might explain the high level of stress tolerance observed in Fiskeby III. In VIGS_Glyma.05G001700 silenced plants at 1D−Fe genes known to be associated with iron stress responses (*TIFY10B*, *NAC9*, *NAC73*, and *scarecrow*) were differentially expressed, with *NAC73* down-regulated under −Fe stress and the remaining genes up-regulated under −Fe stress. In VIGS_Glyma.05G001700 silenced plants exposed to −Fe stress for 7D, over 50% of the DEGs were associated with phosphate deficiency responses, all of which were down-regulated by −Fe stress.

In roots, comparing +Fe and −Fe treated plants identified far more DEGs than in leaves at both timepoints (Figure 2B, Table S5, 1097 DEGs total). There were no genes shared by all four comparisons, and relatively few genes shared by any comparisons, which reinforces the speed and diversity of the soybean stress response. The majority of the DEGs were from plants after 1D−Fe stress (382 DEGs in VIGS_EV_1D and 579 DEGs in VIGS_ Glyma.05G001700_1D). The distribution of DEGs, with more DEGs at 1D−Fe stress than at 7D−Fe stress in both VIGS_EV and VIGS_Glyma.05G001700 suggests Fiskeby III responds quickly to −Fe stress. The greater number of DEGs identified in VIGS_Glyma.05G001700 silenced plants at both 1D−Fe and 7D−Fe confirms that *Glyma.05G001700* plays a role in the Fiskeby III iron homeostasis and stress response. Analyzing all 1,097 DEGs from all four comparisons together generated a heatmap with eight expression clusters (Figure 3B). GO analyses (corrected *p* < 0.05, Table S3) of cluster IR1 reveals genes in this cluster are associated with phosphate deficiency responses (GO:0019375, galactolipid biosynthesis; GO:0080040, response to phosphate starvation; and GO:0006817, phosphate transport) and that the genes in this cluster are almost entirely from VIGS_EV_1D. This supports the phosphate deficiency responses identified by GO analysis in Leaf cluster IL2. After only 24 h of −Fe stress, Fiskeby III was inducing phosphate deficiency responses to re-establish nutrient homeostasis. GO analyses (corrected *p* < 0.05, Table S3) of the four smallest clusters, IR2-IR5, identified lignin catabolism (GO:0046274, IR2) and phloem development (GO:0010088, IR3). Significant GO terms associated with cluster IR6 include four associated with photosynthesis (GO:0009773, electron transport in photosystem I; GO:0015979, photosynthesis; GO0010207, photosystem II assembly; GO:0019684 photosynthesis, light reaction). Examining the individual gene expression profiles in IR6, 11 DEGs shared by VIGS_Glyma.05G001700 at 1D and 7D of −Fe stress are involved with photosynthetic processes. Another five are associated with phosphate (P_i_) deficiency responses, reaffirming the overlapping responses of −P_i_ and −Fe responses in soybean. One gene, *Glyma.04G218900* (homologous to *At5G64840*, *GCN5/ABCF5*) was up-regulated in −Fe at both 1D and 7D of −Fe stress. In Arabidopsis, GCN5 directly regulates key genes controlling Fe and P_i_ homeostasis including FRD3 and PHO2 to maintain a steady state of P_i_ and Fe in cells [36]. Cluster IR7 was associated with plastid organization (GO:0009657) and signal complex assembly (GO:0007172), while cluster IR8 has a single significant GO term (GO:0002679), respiratory burst involved in defense response.

While no genes were differentially expressed in all comparisons in the roots, nine genes were differentially expressed in three of the four comparisons. Among these nine genes are five that are DE in response to −Fe in VIGS_EV_7D, VIGS_Glyma.05G001700_1D, and VIGS_Glyma.05G001700_7D. These five genes include three rubisco genes, a glutaredoxin, and SPX1. Rubisco is the rate-limiting step in photosynthesis. Similarly, the glutaredoxin homolog in Arabidopsis (AT1G64500) is involved in chloroplast movement [37]. SPX1 regulates coumarin biosynthesis under −Fe conditions, resulting in increased fraxin, a glucoside of fraxetin [38]. Our earlier Fiskeby III study identified the upregulation of fraxetin biosynthesis pathways [39]. Increased fraxetin can improve iron availability by extending the pH range for efficient Fe^3+^ reduction, a potentially important approach for improved iron acquisition.

We were also interested in the 486 root genes unique to 1D and the 130 genes unique to 7D of −Fe stress in VIGS_Glyma.05G001700 silenced plants (Table S6). We split the 486 DEGs unique to 1D into up and down-regulated gene lists and conducted go enrichment (Table S6). We identified 16 significantly over-represented GO categories associated with the 225 down-regulated genes, eight related to photosynthesis (GO:0009637, GO:0009657, GO:0009773, GO:0010114, GO:0010207, GO:0010218, GO:0015979, GO:0019684). Interestingly, only 42 DEGs are associated with these GO terms. Examining the 261 up-regulated genes revealed only one statistically significant over-represented GO term, GO:0001666, response to hypoxia. This category accounts for only nine genes. The remaining DEGs, both up and down regulated, are involved in a variety of biological processes, confirming that silencing *Glyma.05G001700* in Fiskeby III has a dramatic and varied response to −Fe stress at 1D.

Among the 130 DEGs unique to 7D, there are no over-represented GO categories associated with the 52 up-regulated genes, but there are four over-represented GO categories associated with the 78 up-regulated genes (GO:0042545, GO:0046274, GO:0006355, and GO:0030643). These GO categories are important for cell wall modification, lignin catabolism, regulating transcription, and cellular phosphate ion homeostasis.

### 2.3. Effect of Time (1D vs. 7D)

The third comparison of our experimental design allows us to examine the effect of time. For this, we compared each construct (either VIGS_Glyma.05G001700 or VIGS_EV) in +Fe or −Fe and compared gene expression differences between 1D and 7D after treatment (Figure 2C). For example, VIGS_Glyma.05G001700, comparing 1D vs. 7D of −Fe stress. Analyzing the effect of 1D−Fe stress compared to 7D−Fe stress revealed more DEGs than the other two analyses. In leaves, the most DEGs (2797 DEGs) were identified in VIGS_EV in +Fe with the majority (2410) being unique to VIGS_EV+Fe (Table S7). Only 755 and 92 DEGs were identified in leaves of VIGS_Glyma.05G001700+Fe and VIGS_Glyma.05G001700−Fe, respectively. Interestingly, while 45% (345 DEGs) of the 755 VIGS_Glyma.05G001700+Fe DEGs are shared with VIGS_EV+Fe, only 6 of the 92 DEGs identified in VIGS_GLyma.05G001700−Fe are shared with VIGS_EV−Fe (Figure 2C). The majority of genes identified in VIGS_EV−Fe (108 DEGs) and VIGS_Glyma.05G001700−Fe (92 DEGs) were unique to their VIGS/iron combination. There are three DEGs common to all four comparisons (*Glyma.08G153600*, *Glyma.08G101500*, *Glyma.09G115100*) and expression profiles are conserved in all four analyses. *Glyma.08G153600* has no Arabidopsis homolog but is thought to encode a transmembrane protein. *Glyma.08G101500* expression increases over time and is homologous to Arabidopsis gene *MRP3*, an ABC-transporter induced by cadmium, nickel, arsenic, cobalt, and lead, but not iron [40]. It is entirely possible that the transporter is induced by iron deficiency, not toxicity. The final conserved gene, *Glyma.09G115100*, is homologous to the Arabidopsis *BRUTUS* (*BTS*) gene, a major iron regulatory gene [41]. Other DEGs of interest include *TIFY10A* homologs (*Glyma.05G027600* and *Glyma.09G071600*), which are down-regulated in VIGS_Glyma.05G001700 exposed to either +Fe or −Fe while *TIFY10B* homologs (*Glyma.01G204400*, *Glyma.11G038600*, and *Glyma.13G112000*) are only down-regulated in VIGS_Glyma.05G001700 exposed to −Fe. The total 2951 DEGs identified in leaves organize into a heatmap with five clusters (Figure 3C). Clusters DL1 and DL5 represent 1642 and 713 DEGs, respectively. GO analysis of the five clusters identified 36 statistically significant GO categories associated with cluster DL1 and 45 GO categories associated with DL5. Only ten of the GO categories in these two clusters are the same and nine of the ten relate to methylation and development (Table S3).

Comparing 1D−Fe stress to 7D−Fe stress in roots revealed the most DEGs of any analysis, 7608 DEGs in total (Figure 2C, Table S8). However, VIGS_EV+Fe and VIGS_Glyma.05G001700+Fe had the most DEGs with 3916 and 4783, respectively. In contrast, VIGS_EV−Fe and VIGS_Glyma.05G001700−Fe had 504 and 2138 DEGs, respectively. As previously, we used the expression profiles to cluster genes, resulting in a heatmap with nine clusters, six of which had over-represented GO terms associated with them (Table S3). Genes in-clusters DR3 and DR5 were associated with DNA replication and methylation. Genes in cluster DR4 were associated with vesicle-mediated transport from the ER to the golgi. Clusters DR7 and DR8 had the most GO terms associated with them, 39 and 45, respectively. The 39 statistically significant GO terms associated with cluster DR7 include three specific to −Fe stress; response to iron starvation (GO:0010106), iron ion transport (GO:0071281), and cellular response to iron ion (GO:0071281). 21 of the remaining GO categories are associated with DNA replication/methylation (9) and defense (12) (Table S3). Genes in cluster DR8 are associated with 38 statistically significant GO terms, including those associated with DNA replication/methylation (9), defense (9) and plant growth and development (8). Among these GO terms are nine terms that were also statistically significant in cluster DR7 (GO:0001510 RNA-methylation, GO:0006412 translation, GO:0008283 cell proliferation, GO:0009611 response to wounding, GO:0009862 systemic acquired resistance, GO:0009867 jasmonic acid mediated signaling, GO:0010200 response to chitin, GO:0031348 negative regulation of defense, and GO:0051567 histone H3-K9 methylation).

## 3. Discussion

### 3.1. Silencing Glyma.05G001700

A previous study by our group revealed that *Glyma.05G001700* plays a role in Fe homeostasis under +Fe conditions [39]. This finding is reaffirmed in the current study where silencing *Glyma.05G001700* resulted in 136 DEGs in leaves and 21 DEGs in roots when comparing VIGS_EV and VIGS_Glyma.05G001700 at both timepoints and both iron conditions. In both leaves and roots, the majority of DEGs (132 and 21) were identified at 7D in +Fe conditions. A single gene, *Glyma.19G001500*, was differentially expressed in all but one treatment comparison (1D +Fe in roots). While silencing impacts *Glyma.19G001500*, it has no effect on its homolog *Glyma.05G001600*, which is located adjacent to the VIGS target gene *Glyma.05G001700*. However, in both leaves and roots in +Fe and −Fe conditions and at both 1D and 7D of −Fe stress, silencing *Glyma.05G001700* results in significant up-regulation of *Glyma.19G001500*. *Glyma.19G001500* is homologous to the Arabidopsis gene *AT3G23590*, also known as *REF4/Med33a/Med5a/b*, hereafter referred to as *Med5a/b*. Med5a/b is a subunit of the Mediator complex which functions as a central coordinator of plant responses to abiotic stresses, with different subunits playing unique roles for different stresses [42]. The Med5a/b subunit regulates transcription as it directly interacts with transcription factors bound to DNA [29]. In Arabidopsis, silencing *Med5a/b* results in increased phenylpropanoid biosynthesis [43]. Flavonoids are a class of phenylpropanoids that accumulate in response to environmental stresses to protect cells from oxidative damage [44,45]. Up-regulation of phenylpropanoids is an important response to −Fe conditions [46]. Phenylpropanoid biosynthesis is important for coumarin synthesis [47]. Our previous study found fraxetin, a coumarin that expands the pH range for efficient Fe^3+^ reduction to improve iron availability, highly up-regulated in Fiskeby III roots after 14 days of −Fe stress [39]. Med5a/b is also known to regulate Plant Defensin 1.2 (PDF1.2, AT5G44420 [30]), which is highly up-regulated in VIGS_Glyma.05G001700 silenced plants compared to EV at both 1D and 7D of −Fe stress (Tables S1 and S2). PDF1.2 is a well-known defensin gene, which responds to ethylene and jasmonic acid to confer resistance to pathogens [48]. It is also induced by ferric sulfate, zinc sulfate, and copper sulfate [49], suggesting PDF1.2 may play a role in mineral homeostasis. This hypothesis is reinforced by the down-regulation of *PDF1.2* in Clark (iron tolerant soybean) after 6 hrs of −Fe stress [50]. Interestingly, there is no soybean homolog for the closely related Arabidopsis gene PDF1.1. In Arabidopsis, PDF1.1 (AT1G75830) shares 100% homology with PDF1.2, but has an additional 2 exons with 100% homology to AT5G44430, which also has no homolog in soybean. It is therefore tempting to hypothesize that Glyma.19G001600 may manifest the roles of these genes in the Fiskeby III −Fe response. This is intriguing given that overexpression of *PDF1.1* in Arabidopsis activates ethylene signaling pathways to induce an iron deficiency response in roots [49]. This proposed role corresponds with the up-regulation of Glyma.19G001500 in response to −Fe conditions.

Given the vast documentation for the role of Med5a/b in Arabidopsis and the up-regulation of the soybean homolog when *Glyma.05G001700* is silenced, it is logical that *Glyma.19G001500* plays an important role in the Fiskeby III iron stress response. However, silencing of *Glyma.05G001600*, which would also silence *Glyma.19G001500*, failed to induce a phenotypic response in Fiskeby III grown under either +Fe or −Fe conditions [39]. Therefore, we hypothesize the IDC QTL on Gm05 may function very similarly to the historical IDC QTL on Gm03 identified in Clark. The Gm03 QTL spans 576 kilobases and 58 candidate genes. However, VIGS analysis of high priority candidate genes in this QTL, including homologs of *AtbHLH038*, *AtbHLH039*, have failed to result in phenotypic changes. Recently, Assefa, J. Zhang, R.V. Chowda-Reddy, A.N. Moran Lauter, A. Singh, J.A. O’Rourke, M.A. Graham and A.K. Singh [14] used a genome-wide association study (GWAS) incorporating 460 diverse soybean lines to investigate the soybean iron deficiency response. For the first time, we demonstrated this historical QTL on chromosome Gm03 is actually four distinct linkage blocks, each with candidate genes contributing to the iron stress response. Silencing of multiple distinct candidate genes is needed to impact iron stress tolerance. Similarly, we hypothesize the Fiskeby III iron stress response is governed by both *Glyma.05G001700* and *Glyma.05G001600* (which would also silence *Glyma.19G001500*) and that silencing both genes simultaneously would significantly alter the Fiskeby III response. Alternatively, Glyma.05G001600 and Glyma.19G001500 may have adopted distinct functions following genome duplication, perhaps for different stress responses. This is the subject of ongoing research in the lab.

### 3.2. Fiskeby III Induces Phosphate Deficiency Responses under −Fe Stress Conditions at 1 Day

Analyses comparing iron stress treatment (+Fe and −Fe) revealed fewer total DEGs in leaves (91) but more DEGs in roots (1097) than comparing silencing treatment (VIGS_EV to VIGS_Glyma.05G001700, Figure 2B, and Tables S3 and S4). In leaves, the majority of the 91 DEGs responding to iron stress treatment were from VIGS_EV_7D. Fifty six of the 62 DEGs were unique to VIGS_EV_7D. However, a heatmap of gene expression patterns finds the majority of these 62 genes are in cluster IL1, which has no statistically significant GO categories (Table S3, Figure 3B). Cluster IL2 of the heatmap, which contains 7 VIGS_EV_7D unique genes along with DEGs shared by both VIGS_EV_7D and VIGS_Glyma.05G001700_7D and genes unique to both VIGS_Glyma.15G001700_1D and VIGS_EV_1D, is enriched for genes associated with cellular phosphate ion homeostasis (GO:0030643), galactolipid biosynthesis and metabolism (GO:0019375 and GO:0009247, GO:0019374), negative regulation of transcription (GO:0045892) and cellular response to -P_i_ (GO:0016036). The statistical significance of these GO categories becomes obvious when the individual annotations of the genes are examined. Three purple acid phosphatases, an *SPX1* gene, and a *phospholipase D* gene are all down-regulated under −Fe conditions. Conversely, a *TIFY10B* transcription factor is up-regulated in −Fe conditions in VIGS_Glyma.05G001700_1D. These changes in gene expression reinforce that Fiskeby III is utilizing phosphate deficiency responses to compensate for the iron-deficient conditions. In addition, these analyses confirm that Fiskeby III was experiencing and responding to −Fe stress within 24 h. Future studies in Fiskeby III will focus on even earlier transcriptional responses to −Fe stress.

The 1097 DEGs identified in roots in response to +Fe vs. −Fe comparisons cluster into eight unique clusters (Figure 3B). Cluster IR1 reaffirms the finding that Fiskeby III utilizes phosphate deficiency responses as the statistically significant GO categories correspond to galactolipid biosynthesis (GO:0019375) and response to −P_i_ (GO:0080040 and GO:0006817). Genes in cluster IR6, are associated with eight GO categories involved with photosynthesis (GO:0009657, GO:0009773, GO:0010114, GO:0015979, GO:0010207, GO:0010218, GO:0009637, GO:0019684) and one associated with detecting biotic stimulus (GO:0009595). These photosynthesis GO categories represent the importance of photosynthesis in the roots and also photosynthetic products being transported to the roots. In cluster IR6 *Glyma.04g218900*, a homolog of *AtGCN5/AtABCF5* is up-regulated by −Fe conditions in VIGS_Glyma.05G001700 silenced plants at 1D and 7D. In Arabidopsis, GCN5 is involved in response to multiple abiotic stresses including maintaining a steady state of P_i_ and Fe [36]. AtGCN5 directly targets 5 genes controlling Fe homeostasis including regulating the histone acetylation of FRD3 [36]. Using all of the Arabidopsis protein homologs of all genes in cluster IR6 as input into StringDB [51], AtGCN5 directly interacts with 12 other proteins (Figure S1), all of which are associated with stress tolerance. Interestingly, although these genes were identified in root tissues, many (GUN5, AOR, PETE1, SCO1, SIG5) are associated with chloroplasts [52,53,54,55,56]. One of the proteins interacting with GCN5 is ABA1, the first committed step in the ABA biosynthesis pathway. Increased ABA alleviates −Fe stress by promoting iron reutilization [57,58]. ABA signaling is also linked to the Mediator complex, with the MED25 subunit the first reported to act in response to ABA [59]. This links the treatment response (VIGS_Glyma.05G001700 vs. VIGS_EV) to the iron stress response (+Fe vs. −Fe).

### 3.3. Days after Stress (DAS)

The majority of the DEGs from this experiment were identified when we compared gene expression after 1D of −Fe stress to expression after 7D of −Fe stress. These comparisons illustrate how Fiskeby III responds to stress over time, the response to a relatively short-term stress exposure (1D) and the response to an extended (7D) stress exposure. In total, 3308 DEGs were identified in leaves and 7662 DEGs were identified in roots. Regardless of the VIGS construct, +Fe plants have more DEGs than −Fe plants. As the plants after 1D and 7D of −Fe stress are the same age, the increase in DEGs in plants moved to new +Fe solutions may reflect the surge of nutrients the 1D +Fe plants access with their new nutrient solutions. In leaves, the largest number of DEGs (2797) are differentially expressed in VIGS_EV+Fe. This is immediately evident from the heatmap (Figure 3C, Table S7), where the leaf clusters are dominated by DL1 and DL5 which reflect VIGS_EV+Fe expression. The expression of genes in DL1 decreased from 1D to 7D of −Fe stress while expression of genes in DL5 increased from 1D to 7D of −Fe stress. Given the large number of DEGs, we primarily focused on identifying over-represented GOs associated with each cluster (Table S3). Overrepresented GO terms associated with DL1 are affiliated with defense processes. Overrepresented GO terms associated with DL5 are affiliated with DNA replication/methylation, cell cycle, and cellular organization, which are characteristic responses of the soybean iron stress response in Clark [13,14]. There are three genes common to all comparisons, including a homolog of *BRUTUS* (*BTS*), which exhibits increasing expression as exposure to −Fe stress increases. In Arabidopsis leaves, BTS functions as an iron sensor to regulate energy metabolism in the shoot, while in roots BTS serves as a negative regulator of Fe homeostasis [41]. POPEYE (PYE) also serves to regulate genes involved in iron transport, storage, and assimilation [60]. While *BTS* is up-regulated as the duration of −Fe stress increases (1D to 7D) in both VIGS_EV and VIGS_Glyma.05G001700 plants exposed to both +Fe and −Fe, *PYE* is up-regulated only in VIGS_Glyma.05G001700 silenced plants exposed to both +Fe or −Fe, but not in VIGS_EV plants. In contrast, *TIFY10*, a transcription factor that responds to multiple abiotic stresses including bicarbonate stress, −P_i_, ozone, drought, high salinity, UV radiation, and cold [61] was down-regulated from 1D to 7D. It is possible *TIFY10* is up-regulated early to induce the Fiskeby III Fe- stress response, then returns to normal levels as Fiskeby III achieves homeostasis under −Fe conditions.

In the roots, a large number of DEGs were identified in both VIGS_EV+Fe (3916) and VIGS_Glyma.05G001700+Fe (4783) (Figure 2 and Figure S1, Table S8). Interestingly, moving to −Fe resulted in fewer gene expression changes than plants that were moved to new +Fe solutions. We hypothesize this may be because plants restrict growth to preserve photosynthetic capacity. Further, the longer both VIGS_Glyma.05G001700 and VIGS_EV plants are exposed to −Fe conditions, they begin to induce responses normally associated with -P_i_ stress. In the DAS root heatmap (Figure 3C), statistically significant GO categories associated with cluster DR9 are involved with galactolipid synthesis. Galactolipid biosynthesis is an important -P_i_ stress response as phosphate groups are scavenged from lipid bilayers [62,63]. While this reinforces the overlap between these two nutrient deficiency responses reported earlier [26], it may further support the hypothesis that in stress conditions Fiskeby III limits growth and causes the plant to reutilize existing resources. Previous studies by our group in the soybean genotype Clark suggest this may be a universal response in soybean [13,23,24,26,64]. However, while Clark and Fiskeby III both inhibit growth in response to stress, they utilize different molecular pathways and the timing of the responses and specific genes involved are unique to each genotype. GO categories associated with cluster DR7 include iron response GO categories response to iron (GO:0010106), iron ion transport (GO:0006826), cellular response to iron (GO:0071281), and DNA replication/methylation (GO:0006260, GO:0051726, GO:0006306), canonical soybean iron deficiency responses.

There was a significant overlap of the statistically significant GO categories in roots and leaves, which is unique for soybean iron deficiency responses. Of the 36 statistically significant GO categories in DL1, 20 were also statistically significant in either DR4 or DR5. Similarly, of the 45 GO categories associated with DL5, 23 were also statistically significant in DR4 or DR5. These overlapping responses could be because these comparisons have not been made before in soybean. Alternatively, the conservation of responses could reflect the differing speed of the response in Fiskeby III compared to Clark (the major soybean line used for −Fe research). The altered timing in Fiskeby III allows us to capture the responses in both leaves and roots simultaneously.

To provide additional validation for the genes identified by RNA-seq, we compared the genes identified in this study to genes identified as differentially expressed in the tolerant soybean line Clark exposed to −Fe stress for 30 min, 60 min, 120 min, 1 h, 6 h, 1 day, 2 days, and 10 days [23,24,26,50]. DEGs were also compared to a recent study by Kholhase et al. [25], which identified DEGs from 18 soybean genotypes exposed to 60 min of −Fe stress. Despite differences in duration of stress exposure and age of plants, 79.2% of the DEGs from leaves and 76.3% of the DEGs from roots from this study were identified as DE in at least one previous −Fe study in soybean (Tables S9 and S10). This provides solid evidence supporting these genes are −Fe responsive and serve to cross-validate the RNA-seq data reported in this study. The genes identified as unique to Fiskeby III further affirm that different soybean lines are utilizing novel genes and networks to confer tolerance to −Fe stress which reinforces the importance of conducting these studies at multiple timepoints and in multiple genotypes.

### 3.4. Conclusions

Our previous work showed soybean has a suite of conserved responses identified in −Fe studies, including iron uptake/homeostasis, defense, DNA replication/methylation, and photosynthesis. The majority of earlier soybean −Fe studies have relied on comparing +Fe-grown plants to −Fe-grown plants and measuring gene expression at different timepoints. These previous studies were also limited to the Clark genotype. This study reaffirms the need to conduct these types of studies on a variety of lines to identify new pathways for crop improvement. This study allowed us to not only compare +Fe to −Fe, but also the silencing of our candidate gene, *Glyma.05G001700*, and how the response of Fiskeby III changes from an initial stress response (24 h) to extended stress (7 days). Through our analyses, we discovered that Fiskeby III invokes all aspects of the canonical soybean iron deficiency response, though the timing of the responses may differ from that of Clark. It was surprising to identify only classic iron uptake/homeostasis genes when comparing 1D vs. 7D of −Fe stress in both leaves and roots. This suggests that upon initial stress exposure Fiskeby III induces a wide array of responses that are subsequently shut off as the period of stress continues.

## 4. Materials and Methods

### 4.1. Plant Growth and VIGS Infection

Fiskeby III seeds were germinated in vermiculite and allowed to grow for 6 days. On day 7, plants were transferred to hydroponics, set up as previously described [14,65,66,67,68]. This hydroponic system mimics calcareous soils of the upper midwestern United States and has been used to identify the same quantitative trait loci in field and hydroponically grown plants [14,67,68]. In brief, the hydroponics solutions were composed of 2 mM MgSO_4_*7H_2_O, 3 mM Mg(NO_3_)_2_*6H_2_O, 2.5 mM KNO_3_, 1 mM CaCl_2_*2H_2_O, 4 mL Ca(NO_3_)_2_*4H_2_O, 0.02 mM KH_2_PO_4_, 542.5 μM KOH, 217 μM DTPA, 20 μM MnCL_2_*4H_2_O, 50 μM ZnSO_4_*7H_2_O, 20 μM CuSO_4_*5H_2_O, 0.2 μM Na2MoO_4_*2H_2_O, 1 μM CoSO_4_*7H_2_O, 1 μM NiSO_4_*6H_2_O, 10 μM H_3_BO_3_. Full nutrient solutions (+Fe) are provided with 100 μM Fe(NO_3_)_3_ while iron deficient (−Fe) solutions are provided with 50 μM Fe(NO_3_)_3_. To reflect the calcareous soil conditions 16.8 g of NaHCO_3_ is added to each hydroponic unit. Finally, all plants are provided a daily solution composed of 10 μmol K_2_HPO_4_, 222 μmol NH_4_NO_3_, and 0.179 μmol H_3_BO_3_. All plants were moved to full nutrient solutions (Fe+, 100 μM Fe(NO_3_)_3_). On day 8, plants were rub-inoculated with either the VIGS_EV vector or the VIGS_Glyma.05G001700 vector. The development of these vectors is described in our previous publication [39]. For rub-inoculation, 25 mg of lyophilized symptomatic BPMV-infected tissue from initial bombardments was added to 500 μL of 50mM potassium phosphate buffer (pH 7.0). Tissue was disrupted using the TissueLyser II (Qiagen^®^, Germantown, MD, USA) to release viral particles into the buffer. Unifoliate leaves of Fiskeby III plants were dusted with carborundum, 20 μL of the inoculum was applied, and leaves were rubbed. Gloves were changed between constructs to ensure no cross-contamination. At the time of inoculation, cotyledons were removed from all plants to force them to utilize nutrients provided by the hydroponics, which results in more consistent gene expression results. Post-VIGS infection, all plants remained in a full nutrient solution. Seven days post inoculation 25% of the plants were moved to iron deficient (Fe− (50 μM Fe(NO_3_)_3_)) conditions and 25% of the plants were moved to new +Fe conditions, the remaining 50% of plants remained in the original solution. Thirteen days post-inoculation, the remaining 50% of plants were moved with half the plants to −Fe conditions and half the plants to +Fe conditions. All plants were harvested 24 h later. At harvest, all leaf tissue and total root systems were collected in separate 50 mL tubes and immediately flash-frozen in liquid nitrogen. Tissue was stored at −80 °C until RNA extraction.

### 4.2. RNA Extraction and Analyses

RNA was extracted from flash-frozen tissue using the Qiagen^®^ RNeasy^®^ Plant Mini Kit (Qiagen^®^, Germantown, MD, USA), following the manufacturer’s instructions. Any contaminating DNA was removed using the Ambion^®^ TURBO DNA-free kit (Ambion^®^, Austin, TX, USA). Qiagen^®^ RNeasy MinElute CleanUp Kits (Qiagen^®^, Germantown, MD, USA) were used to further purify and concentrate samples. Sample quality and purity were measured on an Implen NanoPhotometer N60 (Implen, Munich, Germany). RNA was extracted from four biological replicates and the three with the highest quality were submitted for sequencing. RNA was shipped to the University of Minnesota Genomics Center where quality was again checked using an Agilent BioAnalyzer (Agilent^®^, Santa Clara, CA, USA). Libraries were constructed from 500 ng of RNA using the Illumina TruSeq Stranded mRNA kit. Samples were sequenced on a NovaSeq 6000 (Illumina^®^, San Diego, CA, USA) generating 20 million reads per sample of paired-end reads, each 150 base pairs long. All reads were submitted to the NCBI SRA database under BioProject accession PRJNA878827.

Reads were loaded into the USDA SciNet high-performance computational system for analysis. Read quality was checked using FastQC [69]. VIGS infection was confirmed using FastQ-Screen [70]. This analysis identified four samples (two leaf and two root samples), corresponding to two plants that were not infected with the VIGS construct. Both plants were VIGS_EV plants, one from 1D +Fe stress exposure and one from 7D −Fe stress exposure. All four samples were removed from downstream analyses. On SciNet, reads were mapped to the Williams 82 genome sequence (Glyma.Wm82.a4.v1, Glyma 4.0, https://phytozome-next.jgi.doe.gov/, accessed on 25 December 2022) using hisat2 [71] with default parameters. Uniquely mapped reads were retained using samtools [72]. Data were imported into R-studio [73] for further analysis. The gene feature file (.gff) corresponding to the soybean genome was imported; rtracklayer [74] and GenomicAlignments [75] were used to determine the number of reads aligning to each gene for each sample. The resulting counts file was examined to ensure VIGS_Glyma.05G001700 silenced plants had artificially high gene expression of Glyma.05G001700 and Glyma.19G001600. Even though the genes are silenced, high values for of Glyma.05G001700 and its homolog Glyma.19G001600 were expected as viral reads with a soybean target gene insert will map to the soybean genome (Figure S2). All appropriate samples exhibited high counts, confirming silencing. A modified gff file, one without Glyma.05G001700 or Glyma.19G001600, was uploaded and used for all further analyses. Again, rtracklayer and GenomicAlignments were used to determine the number of reads aligning to each gene for each sample. Data were normalized using the Trimmed Mean of M (TMM) values in the Bioconductor package edgeR [76,77,78]. Using edgeR, normalization factors, tagwise dispersion and differential gene expression were determined. The program ggplots2 was used to visualize replicates to confirm similar gene expression profiles between replicate samples. To identify differentially expressed genes we used a model to account for treatment (EV or VIGS), days after stress (1D or 7D), and hydroponic status (Fe+ or Fe−). Our model grouped samples by type model.matrix (~0 + Group) and contrast statements were used for comparisons. For all comparisons, genes were considered differentially expressed if the false discovery rate (FDR) was <0.05. All leaf samples were normalized together and all root samples were normalized together.

DEGs identified by these analyses were assigned annotations using custom perl scripts as described previously [39]. The primary Gmax v4 proteins were compared to all available *Arabidopsis thaliana* proteins (www.TAIR.org, v10, accessed on 25 December 2022) using BLASTP (E>10–6). The best hit and the most informative hit are reported. Additionally, reported are the gene ontology (GO) annotations associated with each of the Arabidopsis best hits. Over-represented GO terms were identified using custom perl scripts as described in [79] using the GO terms assigned to each gene, a Fisher’s exact test [80] with a Bonferroni correction [81]. Transcription factors were identified using the SoyDB transcription factor database [82].

## Data Availability

Datasets associated with this study can be found in the short-read archive (SRA) database (http://www.ncbi.nih.gov/sra) under BioProject accession PRJNA878827.

## Supplementary Materials

The following supporting information can be downloaded at: https://www.mdpi.com/article/10.3390/ijms24010647/s1.
