# Supplementary material for: Coupling VIGS with Short- and Long-Term Stress Exposure to Understand the Fiskeby III Iron Deficiency Stress Response
Source: Int J Mol Sci. 2022 Dec 30;24(1):647. doi: 10.3390/ijms24010647 (PMC9820625; doi:10.3390/ijms24010647)
Supplement: Supplementary file 1 [file ORourke_Supp Figure S1.pdf › ORourke_Fisk_IJMS_92022_Supp 2/ORourke_Supp Figure S1.pdf]

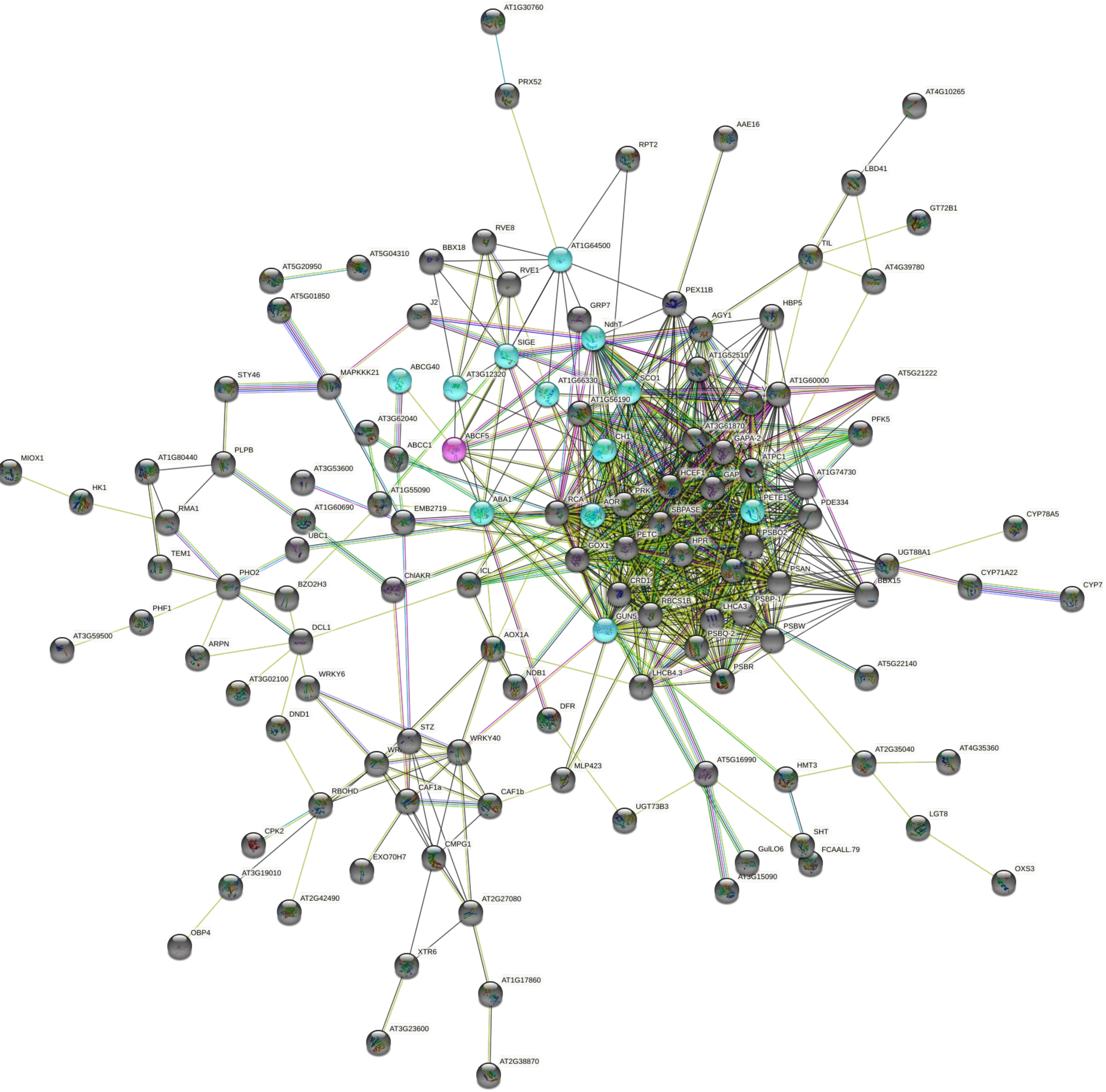

Supplemental Figure S1. IR6 Interaction network. All Arabidopsis homologs of genes associated with cluster IR6 were loaded into String-DB to screen for known interactions. The Arabidopsis gene AtABCF5, also known as AtGCN5, is of particular interest since it is involved in Fe and Pi homeostasis. This gene is highly up-regulated in VIGS-Glyma.05G001700 silenced plants in -Fe conditions at both 1D and 7D. In the String-DB analysis, AtABCF5 (pink) directly interacts with 12 other genes (blue) in the interaction network. These interactions highlight the importance AtABCF5 plays in regulating photosynthesis and possibly ABA biosynthesis in response to -Fe stress.
