# Supplementary material for: Coupling VIGS with Short- and Long-Term Stress Exposure to Understand the Fiskeby III Iron Deficiency Stress Response
Source: Int J Mol Sci. 2022 Dec 30;24(1):647. doi: 10.3390/ijms24010647 (PMC9820625; doi:10.3390/ijms24010647)
Supplement: Supplementary file 1 [file ijms-24-00647-s001.zip › ORourke_Fisk_IJMS_92022_Supp 2/ORourke_Supp Figure S2.pdf]

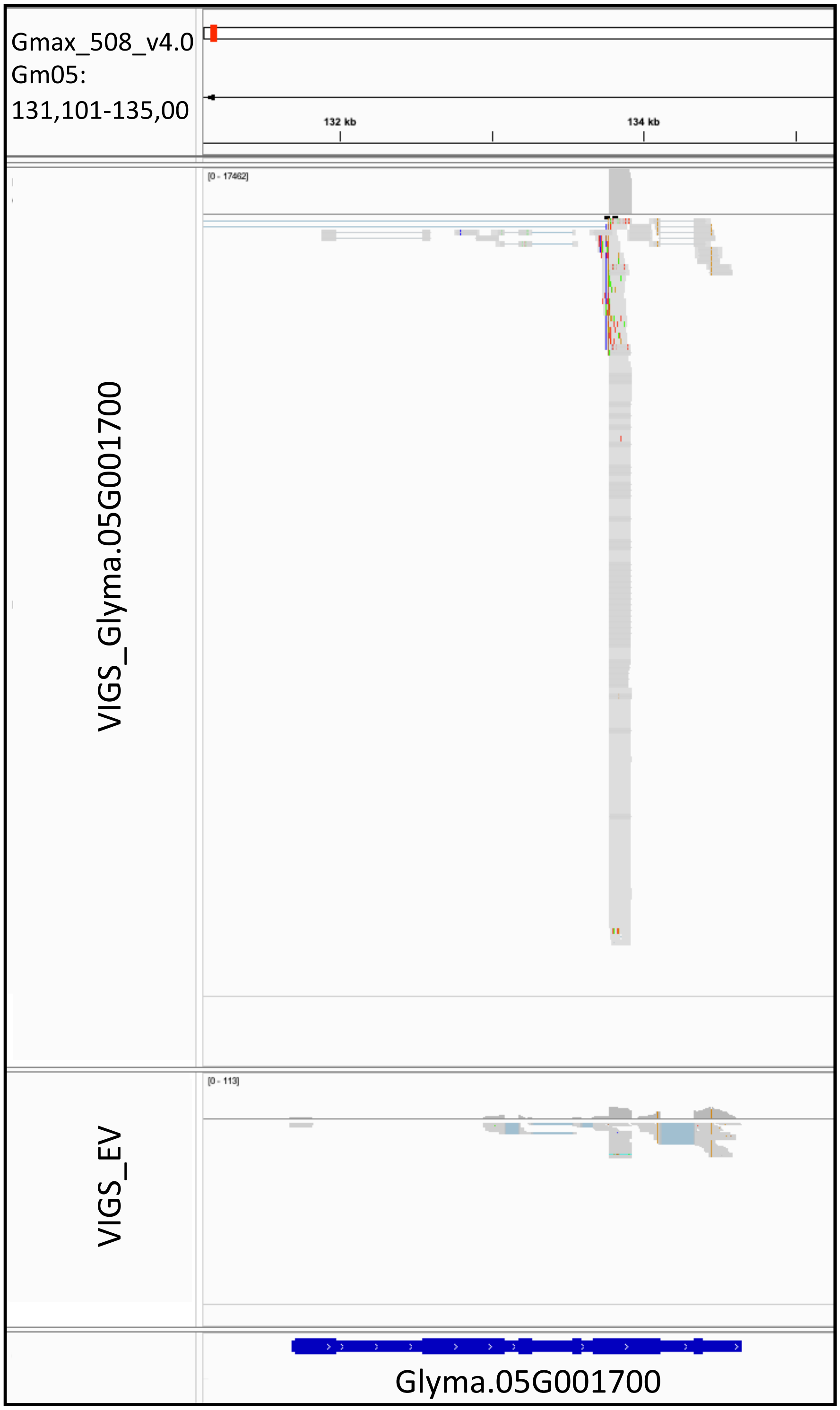


Supplemental Figure S2. Read Alignments in VIGS_Glyma.05G001700 and VIGS_EV. To demonstrate how reads map to the genome we loaded one replicate each of VIGS_EV and VIGS_5G001700 from 1D –Fe into the Integrated Genome Viewer program. These alignments demonstrate the excessive transcripts produced by the virus in the VIGS_5G001700 sample, but not the VIGS_EV sample. This confirms VIGS infection. The lack of additional reads flanking the viral reads in the VIGS_Glyma.05G001700 sample compared to the VIGS_EV sample confirms gene silencing.
